# Pioglitazone stabilizes atherosclerotic plaque by regulating the Th17/Treg balance in AMPK-dependent mechanisms

**DOI:** 10.1186/s12933-017-0623-6

**Published:** 2017-10-30

**Authors:** Yuling Tian, Tao Chen, Yan Wu, Lin Yang, Lijun Wang, Xiaojuan Fan, Wei Zhang, Jiahao Feng, Hang Yu, Yanjie Yang, Juan Zhou, Zuyi Yuan, Yue Wu

**Affiliations:** 1 Department of Cardiology, The First Affiliated Hospital of Xi’an Jiaotong University, 277 West Yanta Road, Xi’an, 710061 Shaanxi China; 2 Department of Vascular Surgery, The First Affiliated Hospital of Xi’an Jiaotong University, Xi’an, 710061 Shaanxi China

**Keywords:** Pioglitazone, AMPK, T cell, Signaling, Atherosclerosis

## Abstract

**Background:**

Pioglitazone (PIO), a thiazolidinediones drug, is a well-known anti-diabetic medicine, but its anti-atherosclerotic effects remain controversial. Thus it is important to investigate the effects of PIO on atherogenesis and the relevant mechanisms.

**Methods:**

For in vitro studies, primary cultured or AMP-activated protein kinase (AMPK) inhibited splenocytes were treated with oxidized low density lipoprotein (ox-LDL) or ox-LDL plus PIO. Percentage of T helper 17 (Th17) and regulatory T (Treg) cells were determined by flow cytometry. Expression of AMPK, interleukin-17 (IL-17) and forkhead box P3 (FoxP3) were detected by Western blots. For in vivo studies, apolipoprotein E–deficient (apoE−/−) mice fed with western diet were treated with PIO or vehicle for 8 weeks respectively. Percentage of Th17 and Treg cells in spleen were measured by immunohistochemical analysis. The atherosclerotic lesions were analyzed using oil red O staining, and collagen types I and III in atherosclerotic lesions were stained by Sirius red. Expression of IL-17 and FoxP3 were determined by quantitative polymerase chain reaction.

**Results:**

In cultured primary splenocytes, PIO dramatically inhibited Th17 and raised Treg. Intriguingly, pharmacological and genetic AMPK inhibitions abolished PIO-induced Treg elevation and Th17 inhibition. Moreover, PIO significantly induced AMPK phosphorylation, decreased IL-17^+^ and increased FoxP3^+^ cells in spleen of apoE−/− mice. Finally, PIO did not alter plaque area, but intriguingly, stabilized atherosclerotic plaque through collagen induction in apoE−/− mice. PIO treatment also improved Th17/Treg balance in atherosclerotic lesions.

**Conclusions:**

PIO exhibits anti-atherosclerotic effects for stabilization of atherosclerotic plaque through regulating the Th17/Treg balance in an AMPK-dependent manner.

## Introduction

Pioglitazone (PIO) belongs to a class of drugs named thiazolidinediones (TZDs), which are the agonists of the nuclear receptor peroxisome proliferator activated receptor (PPAR)-γ. and has been widely used as anti-diabetic drugs. Clinical trials have suggested potential benefits for cardiovascular outcome associated with PIO [1–3]. Several possible mechanisms, including amelioration of atherosclerosis [4–6], and modulating effects on the immune system [7–10], have been proposed. Since the immune response plays a pathogenic role in atherosclerosis [11], the anti-inflammatory properties of TZDs may contribute to its cardiovascular protective effects, including anti-atherosclerosis.

Emerging evidence suggests that T cell adaptive immunity is involved in atherogenesis [11]. For example, T helper 17-cells (Th17), a specific phenotype of CD4^+^ T cells that produce large quantities of interleukin-17 (IL-17), could play an important role in atherogenesis [12]. Another subpopulation of CD4^+^ T cells, regulatory T (Treg) cell expressing a specific transcription factor forkhead box P3 (FoxP3), have been found to suppress the pathogenic immune response of T helper cells against autoantigens or foreign antigens, as well as keep T cell homeostasis [13, 14]. Animal studies have suggested that Treg cells were involved in the development of atherosclerosis [15, 16]. In consistent with these effects of Th17 and Treg, Th17/Treg functional imbalance contributed to atherogenesis in apoE−/− mice [17]. Clinical studies have also proposed a potential role of Th17/Treg imbalance in plaque destabilization and the onset of acute coronary syndrome (ACS) [18, 19]. However, the possible effects of PIO on Th17/Treg balance and the underlying mechanisms remain poorly understood.

AMP-activated protein kinase (AMPK), a fuel-sensing enzyme, is implicated in the regulation of glucose and lipid metabolism [20]. Extensive evidence has suggested that AMPK might be an inflammatory repressor which took part in anti-atherosclerotic process [21–23]. In vitro studies reported TZD-induced activation of AMPK in H-2Kb muscle cells [24], L6GLUT4myc myoblasts [25] (troglitazone) and Swiss 3T3 fibroblasts [26] (troglitazone and pioglitazone). Incubation of isolated Sprague–Dawley rat extensor digitorum longus muscles in medium containing troglitazone significantly increased phospho-AMPK (pAMPK) and phospho-acetyl-CoA carboxylase (pACC), a potent AMPK substrate [26]. Additional in vivo studies confirmed PIO-induced AMPK activation in liver [26, 27], skeletal muscle [26, 27] and adipose tissue [26]. Whether PIO could activate AMPK in T cells remains unknown.

Thus, the aim of the present study was to evaluate whether Th17/Treg balance contributed to the effects of PIO on atherosclerotic lesions and the possible underlying mechanisms related to AMPK activation.

## Materials and methods

### Animals

Apolipoprotein E–deficient (apoE−/−) mice (B6.129P2-apoEtm1Unc strain) were purchased from Model Animal Research Center of Nanjing University and kept in a temperature-controlled facility on a 12 h light/dark cycle with free access to food and water. All mice were weaned at 4-week-old and fed normal chow to 6-week-old. Then they were fed on western diet (21% fat and 0.15% cholesterol) for 8 weeks when early atherosclerotic lesions such as fatty streak could be induced in the root of aorta. 16 and 6 mice were randomly selected for in vivo and in vitro studies, respectively. All animal experimental protocols were approved by the institutional ethics committee for animal experiments of Xi’an Jiaotong University. All surgical and experimental procedures were carried out in accordance with the Guide for the Care and Use of Laboratory Animals of National Institutes of Health revised in 2011.

### Animal treatment

PIO was provided by Hengrui Medicine, Ltd. (Jiang Su, China). It was dissolved in 0.5% sodium carboxy methyl cellulose. Mice at the age of 6 weeks were divided into two groups (n = 8) randomly and treated with vehicle or PIO (20 mg/kg/day) orally by gastric gavage for 8 weeks.

### Analysis of atherosclerotic lesions in aorta

At the end of the experiment, all mice were sacrificed. The root of the aorta was dissected under a macroscope and frozen in optimal cutting temperature embedding medium for serial 6 μm cryosectioning, which covered 500 μm of the root. The atherosclerotic lesions were detected using oil red O staining. The cross-sectional surface area of atherosclerotic lesions and vessel area were quantified. Collagen types I and III in atherosclerotic lesions were stained by Sirius red and the sections were analyzed by polarization microscopy.

### Immunohistochemical analysis

Immunohistochemical analysis was performed as described previously [21]. The spleen was collected and frozen in optimal cutting temperature embedding medium for serial 6 μm cryosectioning. Immunohistochemical analysis was performed against IL-17 (BD PharMingen 1:100) and FoxP3 (eBioscience 1:200) using 6 spleen sections per mouse to measure the percentage of Th17 and Treg cells in spleen. The percentages of the total area of the immunohistochemically positive cells were assessed using a microscopy image analysis system. A total of three fields was randomly chosen and analyzed at 400 × magnification.

### Cell culture

To examine the effect of PIO on Th17 and Treg, we used primary cultured splenocytes obtained from the remaining six mice. Briefly, splenocytes were isolated with a nylon cell strainer and red blood cells was lysed and subsequently washed. Splenocytes were cultured (1.5 × 10^6^/mL) at 37 °C under 5% CO_2_ 95% air in RPMI 1640 medium (Gibco-BRL) supplemented with 10% fetal bovine serum (Gibco-BRL) and 1% penicillin–streptomycin (North China Pharmaceutical Co., Ltd). In all cultured splenocytes, 2 μg/mL oxidized low density lipoprotein (ox-LDL) produced from our lab was added for antigenic stimulation [28]. 2 nmol/mL PIO were added separately to cultured splenocytes for 12 h incubation.

### Quantitative PCR

RNA isolation and quantitative real-time PCR were performed as described previously [29].

### Flow cytometry

The cultured splenocytes were stained with FITC-labeled anti-CD4 (BD PharMingen) and PE-labeled IL-17 (BD PharMingen) to determine the presence of CD4^+^IL-17^+^ cells. FITC-labeled rat IgG2b and PE-labeled rat IgG1 (BD PharMingen) were used for isotypic control. CD4^+^CD25^+^FoxP3^+^ cells were detected by FoxP3 intracellular staining using mouse regulatory T cell staining kit (eBioscience) according to manufacturer’s protocol. Briefly, cells were counterstained with FITC-labeled anti-CD4 (RM4-5) and PE-labeled anti-CD25 (PC61.5). After incubation of 30 min, cells were fixed, washed and resuspended in a permeabilization solution. The permeabilized cells were stained with APC-labeled anti-FoxP3 (FJK-16s). FITC-labeled rat IgG2b, PE-labeled rat IgG1 and APC-labeled rat IgG2a were used for isotypic control. Stained cells were analyzed on a FACScan flow cytometer, using CellQuest software (Becton–Dickinson,). Procedures were performed as described previously [19].

### Western Blot (WB)

Cells or spleen tissues were homogenized in RIPA lysis buffer (Santa Cruz), and protein contents were measured using the Bradford (BCA) assay (Pierce). Anti-phospho-AMPK (CST), AMPK (CST), Foxp3 (CST), IL-17 (BD), and Gapdh (Santa Cruz) was used. Samples were subjected to Western blot as described previously [29].

### Statistical analysis

The results are expressed as mean ± standard error of mean (SEM). One-way analysis of variance (ANOVA) was performed to compare the difference among groups. *P* < 0.05 were considered statistically significant. All statistical analysis was performed using SigmaStat3.1.

## Results

### PIO attenuates ox-LDL-enhanced IL-17 expression in cultured primary splenocytes

**Fig. 1:**
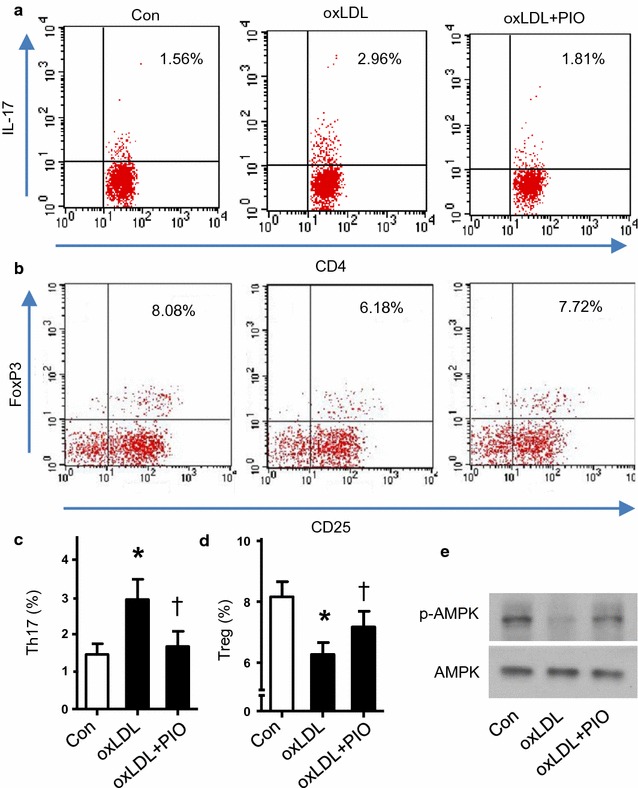
Effects of PIO on Th17/Treg balance and AMPK activation in cultured primary splenocytes. **a**, **b** Representative flowcytometric results of Th17 and Treg cells, respectively. Numbers represent the percentage of cells in the quadrants. **c**, **d** Quantitative analysis of Th17 and Treg population among the 3 groups respectively. *P < 0.05 vs Con; ^†^ *P* < 0.05 vs ox-LDL. **e** Representative western blot analysis of pAMPK and AMPK in splenocytes treated with or without ox-LDL and PIO

We first investigated whether PIO could influence Th17 cell population in cultured splenocytes isolated from apoE−/− mice. Th17 cells were determined by flowcytometry evaluating the CD4^+^IL17^+^ cells. Ox-LDL, a well-known atherosclerotic risk factor, is widely used as a classic stimulus in vitro. As depicted in Fig. 1a, c, ox-LDL significantly enhanced IL-17 expression in cultured splenocytes, but PIO dramatically attenuated the IL-17 expression, indicating that PIO blocked Th17 cell formation induced by ox-LDL.

### PIO rebalances Th17/Treg in vitro

T helper cells including Th17 cells can also differentiate into regulatory T cells (Treg). Thus, we detected the Treg cell population (CD4^+^CD25^+^FoxP3^+^ cells) as well. As shown in Fig. 1b, d, ox-LDL dramatically decreased Treg cells, which can be blocked by PIO. These data suggested that PIO improved the balance of Th17/Treg disturbed by ox-LDL in vitro.

### PIO rebalancesTh17/Treg by AMPK activation

Activation of AMPK has been known to suppress inflammation [21], and is related to increased Treg in inflammatory diseases [30]. Therefore we studied whether PIO altered AMPK activation by evaluating the phosphorylation of AMPK at Thr-172. Intriguingly, ox-LDL inhibited AMPK phosphorylation, but PIO blocked its inhibition (Fig. 1e), indicating that PIO induced AMPK activation.

**Fig. 2:**
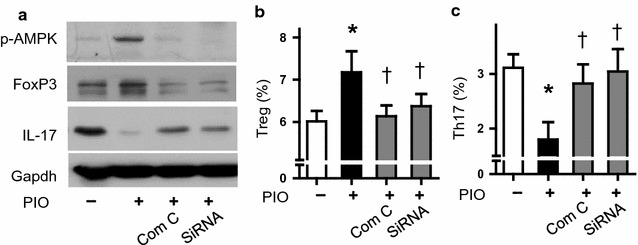
Effects of AMPK on Th17/Treg balance. **a** Representative western blot analysis of pAMPK, FoxP3 and IL-17 in splenocytes with or without AMPK inhibition. Gapdh was used as a loading control. **b**, **c** Quantitative analysis of Th17 and Treg populations using flow cytometry, respectively. *P < 0.05 vs Con; ^†^ *P* < 0.01 vs PIO only

To further assess the function of AMPK, we used compound C (Com C, a potent AMPK inhibitor) and AMPK siRNA to test whether AMPK activation is required for PIO-induced Th17/Treg rebalance. As shown in Fig. 2a, both pharmacological and genetic means abolished PIO-induced AMPK activation, FoxP3 induction and IL-17 inhibition. Moreover, flow cytometry data showed that Compound C and AMPK siRNA blocked PIO-induced Treg formation and Th17 inhibition (Fig. 2b, c). These data established that AMPK activation was required for PIO-mediated Th17/Treg rebalance.

### PIO rebalances Th17/Treg and AMPK activation in vivo

**Fig. 3:**
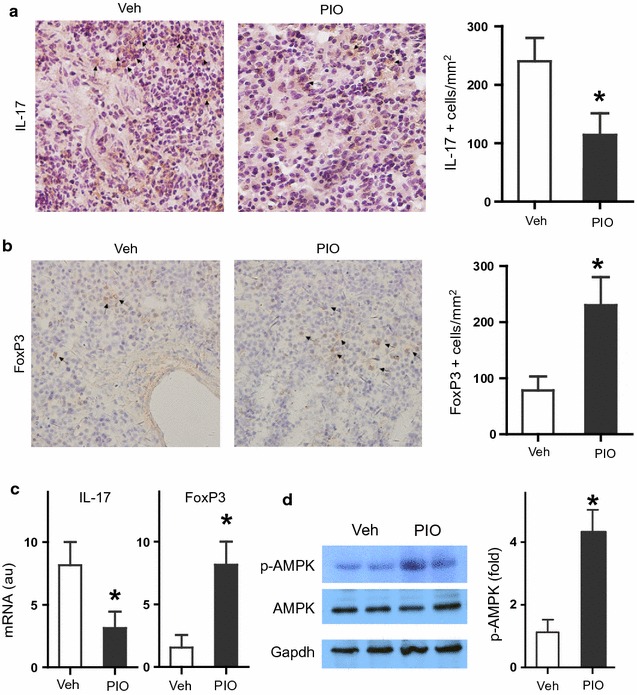
Effects of PIO on Th17/Treg balance, IL-17/FoxP3 expression, and AMPK activation in spleen. **a** Representative photos and statistic analysis of immunohistochemical staining of IL-17^+^ cells. **b** Representative photos and statistic analysis of immunohistochemical staining of FoxP3^+^ cells. **c** mRNA expression of IL-17 and FoxP3. **d** The pAMPK, AMPK and Gapdh were detected by Western blots. *P < 0.05 vs Veh

To determine the effects of PIO on Th17 cells and Treg cells in vivo, we examined the expression level of IL-17^+^ cell and FoxP3^+^ cell in spleen from mice treated with PIO or vehicle. Figure 3a, b showed decreased expression of IL-17^+^ splenocytes and increased expression of FoxP3^+^ splenocytes in PIO-treatment mice than control. Moreover, PIO also decreased IL-17 mRNA and increased FoxP3 mRNA level in vivo (Fig. 3c), indicating PIO regulated the Th17/Treg balance in vivo.

To determine the effects of PIO on AMPK activation in vivo, we detected (pAMPK) and AMPK in spleen from mice treated with PIO or vehicle. Figure 3d showed the increased level of pAMPK in PIO-treatment mice than control, but the level of AMPK and Gapdh didn’t change in PIO-treated mice, indicating PIO also induced AMPK activation in vivo.

### PIO stabilizes the atherosclerotic lesions in vivo

**Fig. 4:**
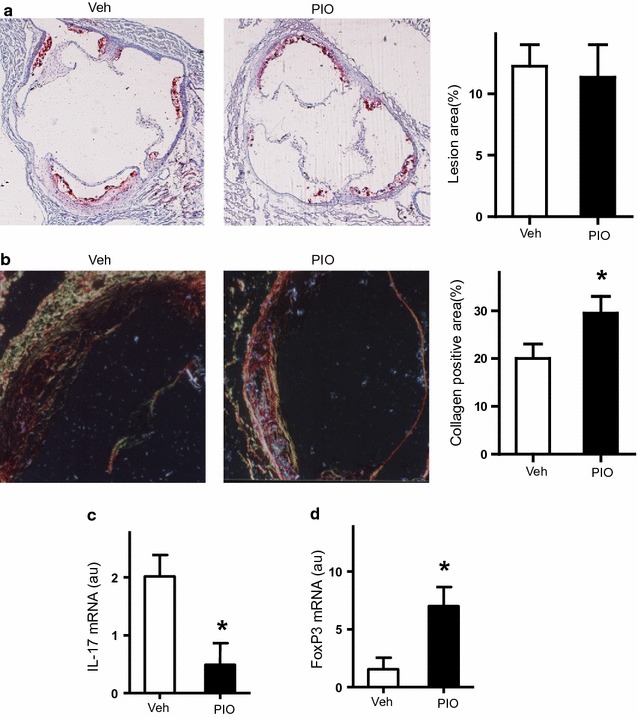
Effects of PIO on atherosclerotic lesions. **a** Representative photos and statistic analysis of the area of atherosclerotic lesions in the aortic root. **b** Representative photos and statistic analysis of collagen content in the aortic root. **c**, **d** mRNA expression of IL-17 and FoxP3 in atherosclerotic plaque, respectively. *P < 0.05 vs Veh

Since Th17/Treg balance is closely related to atherosclerotic lesions, we detected the area of atherosclerotic lesions in the aortic root of apoE−/− mice treated with PIO or vehicle. Figure 4a–c showed that atherosclerotic lesions in PIO-treated mice were similar to those in control mice (*P* > 0.05).

Next, we detected the plaque stability by evaluating the content of collagen. Intriguingly, the collagen content in PIO-treated mice (30.12 ± 2.08%) was significantly increased compared with control mice (21.07 ± 2.73% P = 0.001), suggesting PIO stabilized atherosclerotic lesions in vivo (Fig. 4a, b).

### PIO regulates the Th17/Treg balance in atherosclerotic lesions

T cell subgroups have been well-established in atherosclerotic lesions, especially Th17 and Treg cells [17]. Thus, we determined the mRNA expression of IL-17 and FoxP3 in mouse atherosclerotic lesions. As shown in Fig. 4c, d, PIO, not the vehicle, dramatically enhanced FoxP3 expression and attenuated IL-17 expression in atherosclerotic lesions. It demonstrated that PIO regulated the Th17/Treg balance in atherosclerotic lesions.

## Discussion

Our study found that (1) PIO stabilized atherosclerosis lesions in apoE−/− mice; (2) RebalancingTh17/Treg was essential for anti-atherosclerotic effects of PIO; and (3) AMPK activation was required for this PIO-regulated Th17/Treg rebalance.

Our finding of stabilized atherosclerotic effects in mice was partly consistent with the reported vascular effect of PIO in animal and human studies. For example, Game et al. [31] found 12-week of PIO treatment could reduce atherosclerotic lesions in mice. In type 2 diabetes patients with 18-month treatment of PIO, the progression of carotid artery intima-media thickness [32] and coronary atherosclerosis [3] were both decreased compared with patients treated with glimepiride, an oral anti-diabetic medicine to control blood sugar levels. Although some human studies have shown that the risk of heart failure increased [33], and left ventricle diastolic function reduced [34] in PIO users, recent studies found that PIO therapy was associated with lower risks of non-fatal myocardial infarction, non-fatal stroke and cardiovascular death in patients with abnormal glucose metabolism [33, 35]. These beneficial impacts of PIO on atherosclerotic diseases may be related to its atherosclerotic stabilizing effect.

Since Th17/Treg balance plays an important role in adaptive immunity, and has a close relationship to atherogenesis [17], we studied the effects of PIO on Th17/Treg balance. We found a decreased level of IL-17^+^ cell and an increased level of FoxP3^+^ cell in spleen of mice treated with PIO. In cultured splenocytes treated with PIO, we found decreased mRNA level of IL-17 and increased mRNA level of FoxP3. Flow cytometry analysis showed decreased percentage of CD4^+^IL-17^+^ cells and increased percentage of CD4^+^CD25^+^FoxP3^+^ with PIO treatment. Our work was consistent with a previous report showing that TZDs could enhance Treg generation in vivo and in vitro [36]. As activators of the nuclear receptor PPAR-γ, TZDs might inhibit atherosclerosis through PPARγ-dependent and PPARγ-independent mechanisms [37, 38]. It might be involved in T cell adaptive immunity through modulation of the level of Treg and Th17 populations.

We next studied the underlying mechanisms in PIO-regulated Th17/Treg balance. As expected, PIO stimulated AMPK activation in splenocytes. Pharmacological and genetic silence of AMPK abolished PIO-induced FoxP3 induction and IL-17 inhibition and PIO-induced Treg formation and Th17 inhibition. This was consistent with a previous study proposing immune regulation effects of AMPK activation on Th17/Treg balance in inflammatory bowel disease [39].

Previous studies have established the upstream signaling of AMPK [40]: liver-kinase B1(LKB1) and Ca(2+)/calmodulin-dependent kinase kinase 2 (CaMKKβ), which induce phosphorylation at threonine (Thr-172) and activate AMPK. AMP/ATP ratio can also induce AMPK phosphorylation and activation. AMPK has several known downstream targets. Retinoic acid-related orphan receptor γt (RORγt), signal transducers and activators of transcription (STAT), and Foxp3 are key transcription factors for T cell [41]. Some of them are potential AMPK downstream targets to tip the Th17/Treg balance. Further studies are warranted to investigate the AMPK-mediated regulation of transcription factors in PIO-induced Th17/Treg rebalance.

## Conclusions

The present study confirmed the effects of PIO on stabilization of atherosclerotic plaque in apoE−/− mice. Modulation of T cell adaptive immunity involving Th17/Treg balance was the underlying mechanisms. And AMPK activation was required for this PIO-regulated Th17/Treg rebalance.

## Abbreviations

PIO: pioglitazone
ox-LDL: oxidized low density lipoprotein
Th17: T helper 17
Treg: regulatory T
AMPK: AMP-activated protein kinase
IL-17: interleukin-17
FoxP3: forkhead box P3
apoE−/−: apolipoprotein E–deficient
TZDs: thiazolidinediones
PPAR: peroxisome proliferator activated receptor
ACS: acute coronary syndrome
WB: Western Blot
pAMPK: phospho-AMPK
pACC: phospho-acetyl-CoA carboxylase
LKB1: liver-kinase B1
CaMKKβ: Ca(2+)/calmodulin-dependent kinase kinase 2
Thr: threonine
STAT: signal transducers and activators of transcription
RORγt: retinoic acid-related orphan receptor γt
